# [Retracted] Exosomal miR‑130a‑3p promotes the progression of differentiated thyroid cancer by targeting insulin‑like growth factor 1

**DOI:** 10.3892/ol.2023.14017

**Published:** 2023-08-22

**Authors:** Guang Yin, Wencheng Kong, Sixin Zheng, Yuqiang Shan, Jian Zhang, Rongchao Ying, Hao Wu

Oncol Lett 21: 283, 2021; DOI: 10.3892/ol.2021.12544

Following the publication of this paper, a concerned reader drew to the Editor's attention that there appeared to be quite an extensive overlap of data (albeit with a different microRNA being reported on) with the following article, which contained an entirely different set of authors based at different research institutions: Li S, Zhang S, Yang W, Li F and Long H: Diagnostic value of exosomal miR-148a-3p in the serum of patients with differentiated thyroid cancer. Clin Lab: 67, 2021. The Editorial Office conducted an independent investigation of this matter, and were able to substantiate the reader's claims; moreover, a paper submitted to and accepted in the journal Kaohsiung J Med Sci over a similar time period, which did feature some of the same authors as the abovementioned Oncol Lett paper, likewise contained several of the same data.

Owing to the fact that a large number of data in this paper had also been published in two other articles that were accepted at approximately the same time, the Editor of *Oncology Letters* has decided that this paper should be retracted from the journal. he authors were asked for an explanation to account for these concerns, but the Editorial Office did not receive a reply. The Editor apologizes to the readership for any inconvenience caused.

